# Passage of *Borrelia burgdorferi* through diverse Ixodid hard ticks causes distinct diseases: Lyme borreliosis and Baggio-Yoshinari syndrome

**DOI:** 10.6061/clinics/2018/e394

**Published:** 2018-11-06

**Authors:** Carmen Silvia Molleis Galego Miziara, Virginia Aparecida Gelmeti Serrano, Natalino Yoshinari

**Affiliations:** I Neurologia, Hospital das Clinicas HCFMUSP, Faculdade de Medicina, Universidade de Sao Paulo, Sao Paulo, SP, BR; II Reumatologia, Hospital das Clinicas HCFMUSP, Faculdade de Medicina, Universidade de Sao Paulo, Sao Paulo, SP, BR

**Keywords:** Lyme Disease, Baggio-Yoshinari Syndrome, *Borrelia burgdorferi*, Neuroborreliosis, Tick-Borne Diseases

## Abstract

Baggio-Yoshinari syndrome is an emerging, tick-borne, infectious disease recently discovered in Brazil. This syndrome is similar to Lyme disease, which is common in the United States of America, Europe and Asia; however, Brazilian borreliosis diverges from the disease observed in the Northern Hemisphere in its epidemiological, microbiological, laboratory and clinical characteristics. Polymerase chain reaction procedures showed that Baggio-Yoshinari syndrome is caused by the *Borrelia burgdorferi sensu stricto* spirochete. This bacterium has not yet been isolated or cultured in adequate culture media. In Brazil, this zoonosis is transmitted to humans through the bite of *Amblyomma* and *Rhipicephalus* genera ticks; these vectors do not belong to the usual Lyme disease transmitters, which are members of the *Ixodes ricinus* complex. The adaptation of *Borrelia burgdorferi* to Brazilian vectors and reservoirs probably originated from spirochetes with atypical morphologies (cysts or cell-wall-deficient bacteria) exhibiting genetic adjustments, such as gene suppression. These particularities could explain the protracted survival of these bacteria in hosts, beyond the induction of a weak immune response and the emergence of serious reactive symptoms. The aim of the present report is to note differences between Baggio-Yoshinari syndrome and Lyme disease, to help health professionals recognize this exotic and neglected zoonosis.

In 1975, in Old Lyme, Connecticut, United States of America (USA), an outbreak similar to juvenile rheumatoid arthritis was registered whose occurrences were mostly associated with erythema migrans (EM), which is a skin rash that radiates from the site of a tick bite. The outbreak was investigated by the physicians Allen C. Steere and Stephen E. Malawista and led to the discovery of a new zoonosis known as Lyme disease (LD) 1. In 1982, Willy Burgdorfer identified a new *Borrelia* species in ticks, which was later designated *Borrelia burgdorferi* and found to be the etiologic agent of LD 2.

The *Borrelia burgdorferi* spirochete is transmitted to vertebrate hosts by *Ixodes* genus ticks in the USA, Europe and Asia, causing multiple clinical manifestations in both adults and children. However, diagnosis is more difficult in children because the characteristics of borreliosis disease are very similar to those of common childhood illnesses 3.

In Brazil, the first cases involving LD-similar clinical manifestations were described in 1992, in siblings from the municipality of Cotia, State of São Paulo, who were bitten by ticks and developed EM, arthritis and positive serology against *Borrelia burgdorferi* 4.

An important new epidemiological discovery made by researchers from the Faculdade de Medicina da Universidade de São Paulo (FMUSP) was that the transmission of this disease occurred via the bites of *Amblyomma* and *Rhipicephalus* genera ticks, which are vectors not belonging to the *Ixodes ricinus* complex 4,5. This intriguing finding had never previously been reported in the medical literature. In this original and never-before-described context, the zoonosis found in Brazil was named Brazilian LD like syndrome, or Baggio Yoshinari syndrome (BYS). The latter nomenclature seems to be more appropriate because LD and BYS present many distinguishing features 4,5.

BYS is currently defined as an emerging infectious disease, originally identified in Brazil and perhaps also present in other South American countries, that is caused by the *Borrelia burgdorferi* spirochete in its atypical morphological presentation and is transmitted by ticks not belonging to the *Ixodes ricinus* complex. Clinically, BYS diverges from LD in that it can progress with recurrent and/or reactive symptoms that are difficult to diagnose and treat.

The biodiversity and peculiar climate of Brazil probably contributed to the emergence of a borreliosis with specific microbiological, laboratory and clinical features different from those observed in the Northern Hemisphere. A spirochete with specific morphological and genetic characteristics that has adapted to survive in the country's ecologically biodiverse conditions may have originated via the passage of *Borrelia burgdorferi* through the digestive tracts of Brazilian exotic ticks. The etiological agent of Brazilian borreliosis has not yet been isolated or cultured, although it can be identified via immunohistochemical methodology using focus-floating microscopy 6 and molecular methods 7,8, which have demonstrated the existence of *Borrelia burgdorferi sensu stricto* spirochetes.

When cultured under adverse conditions of pH or temperature, or in the presence of antibiotics, *Borrelia* bacteria develop morphological alterations similar to elongated bacteria (bacteroids), dense bodies suggestive of *Chlamydia* bacteria or the appearance of bacteria deprived of cell walls resembling *Mycoplasma* 8-11. These structural alterations may be associated with the loss or inactivation of genes (mutations) that regulate the synthesis of flagella or lipoproteins of the outer membrane surface proteins (Osps) 12,13. These genetic and morphological changes probably affect important molecular interactions between *Borrelia burgdorferi* and its vertebrate reservoir hosts and invertebrate vectors (ticks), especially when they involve the expression or suppression of genes related to the synthesis of flagellin and Osps 13. The occurrence of ticks not belonging to the *Ixodes ricinus* complex in Brazil explains all the clinical and laboratorial particularities observed in BYS.

Electron microscopy analysis of BYS patient blood revealed the presence of non-motile structures similar to those reported as spirochetes in cystic forms. Importantly, *Borrelia* bacteria with this presentation are difficult to culture and isolate in Barbour Stoenner-Kelly modified (BSK) culture media, as observed in Brazil 14.

Borreliosis has been studied at FMUSP for almost 30 years in an attempt to identify LD in Brazil. Based on the description of many patients originating from all across the country, many differences have been observed between the zoonosis occurring in Brazil and that reported in the Northern Hemisphere. Due to these differences, Brazilian borreliosis has been referred to an LD-like illness, infective-reactive LD-like syndrome and, finally, as Baggio-Yoshinari syndrome.

Brazilian researchers have propose that the main causative factor responsible for these differences is related to passage of *Borrelia burgdorferi* through exotic Ixodid ticks not belonging to the *Ixodes* genus, which includes the tick species *Rhipicephalus sanguineus*, *R. microplus*, *Amblyomma cajennense*, and *Dermacentor nitens*. According to our research, the passage of *Borrelia burgdorferi* through Ixodid ticks not belonging to the *Ixodes ricinus* complex and Brazilian reservoirs mainly consisting of domestic animals caused the appearance of *Borrelia* adapted to survive in our country's conditions, resulting in the emergence of the exotic zoonosis referred to as BYS.

The clinical manifestations of BYS are similar to those of LD. EM occurs at the tick bite site during the acute phase in approximately 50% of cases. Articular, neurological, cardiac or ophthalmic complications are observed in the secondary disease stage. In the tertiary phase, chronic arthritis, skin lesions similar to those observed in scleroderma and degenerative neuropathy can be observed. Recurrence of symptoms, even in previously antibiotic-treated patients, is an important distinguishing feature of BYS.

An analysis of patients with BYS confirmed the relevance of epidemiological interrogations conducted at anamnesis, requests for information about bites or contact with ticks, and visits to risk areas. In addition to domestic animals (such as dogs, horses, or bovines), wild animals (such as small rodents, marsupials and capybaras) are implicated in the disease transmission cycle. An epidemiological survey must include the profession of the suspected patient, since infection risk is high in humans who visit forested or grassy areas. It is important to remove a tick before 24h after tick fixation to the skin to avoid disease installation.

The main clinical presentations involving the nervous system include the triad of meningitis, cranial neuritis and peripheral neuropathy. Ocular involvement and psychiatric complaints are two additional relevant features described in 36.7% and 20% of patients, respectively, from a group of 30 patients with adult neuroborreliosis 4. Ocular disorders can present as lesions of cranial neuritis (diplopia, eyelid ptosis) or as intrinsic eye inflammation, including optical nerve damage, papilledema, uveitis and chorioretinitis. Encephalitis either alone or associated with meningitis is another severe presentation of neuroborreliosis that is difficult to treat 15; Guillain-Barré syndrome (GBS) may also be observed. Lymphomononuclear meningitis, which is reported in approximately 30% of BYS cases 4,16 was first described in Brazil in 1996 17.

Studies on Brazilian neuroborreliosis are important because nearly 35% of BYS cases have a neurological clinical presentation, and there are few papers reporting neuroborreliosis in children. The relevance of this topic was seen in a Brazilian study in which the prevalence of anti-*Borrelia burgdorferi* antibodies was observed in 6.2% of children with acute exanthema, with higher seasonal expression in summer and autumn 18.

Arthritis of large joints (mainly of the knee) occurs in nearly 30–35% of BYS patients. The crisis of arthritis can last for a few weeks and may be recurrent. In general, the joint effusion is enormous, and the synovial fluid has an inflammatory character. A small percentage of patients may progress to chronic arthritis, similar to rheumatoid arthritis, a fact in which the use of corticosteroids or immunosuppressive drugs may be recommended. Myositis is a rare condition observed in Brazilian borreliosis. Heart involvement is rare; patients may show different degrees of atrium-ventricular block, but the use of a pace maker is generally unnecessary 5.

Serological diagnostic tests (ELISA and western blotting) performed to detect antibodies against *Borrelia burgdorferi* in Brazil using a sonicated crude extract of *Borrelia burgdorferi* of North American origin present low sensitivity and specificity. The CDC criteria for serologic diagnosis of LD adopted in the USA, based on an initial ELISA or IFA screening test, followed by western blotting examination in doubtful cases, fail in Brazil. This failure can be explained by the previously noted fact that *Borrelia burgdorferi* sensu stricto found in Brazil expresses minor amounts of antigens, including exhibiting an absence of flagellin filament and outer membrane surface proteins. Thus, FMUSP performs ELISA and western blotting simultaneously in suspected cases, despite the risk of false-positive results. Diagnosis of BYS is difficult in the absence of EM, the distinguishing feature of BYS. Most cases are diagnosed on an epidemiological and clinical basis, after exclusion of other diseases that cause symptoms related to those of BYS. Serological tests can be helpful, but alone, they do not provide a diagnosis of BYS.

The first polymerase chain reaction (PCR) test conducted in Brazil employing primers derived from the *flgE* gene was fundamental to understanding most of the questions related to the study of borreliosis in this country. First, this molecular procedure revealed 99% homology to the *Borrelia burgdorferi* sensu stricto gene *flgE* in samples from humans with BYS, ticks of the genus *Rhipicephalus* and the blood of domestic animals such as horses and bovines. Furthermore, sequencing of the DNA products derived from PCR targeting the *flgE* gene showed that nucleotide alignment was identical for all samples, with the exception of differences in two bases, always at the same site in the *flgE* gene structure. This research demonstrated that *Borrelia burgdorferi* circulating in patients with BYS, ticks and animal reservoirs are genetically identical, contributing to the elucidation of the transmission cycle of this borreliosis in Brazil.

The diagnostic criteria for BYS adopted at FMUSP comprise both major and minor parameters. The major parameters include (a) positive epidemiology, such as a tick bite episode and/or contact with wild or domestic animals infested with ticks, or a visit to a risk area; (b) the presence of EM or systemic manifestations (articular, neurological, cardiac or ocular); and (c) positive serology for *Borrelia burgdorferi*. The minor parameters include (a) relapse episodes and (b) symptoms compatible with chronic fatigue syndrome (CFS). A hypothesis of BYS is considered in the presence of three major parameters or two major parameters plus two minor parameters.

Treatment of BYS must start as soon as possible because a delay in drug administration longer than three months after disease onset can result in further difficulties in approximately 75% of cases, including disease recurrence or the appearance of reactive symptoms 5. Patients in the acute stage of BYS, exhibiting the presence of local EM without evidence of disease dissemination, are treated with antibiotics for a short period of time (generally less than one month). If blood dissemination of bacteria is noted, including flu-like symptoms, we recommend maintenance of antibiotics for three months. Additionally, in an attempt to avoid an outbreak of autoimmune symptoms, the use of immunomodulatory drugs such as hydroxychloroquine or sulfasalazine is recommended, sometimes for a long period of time. BYS patients identified at secondary or tertiary stages are treated with antibiotics and immunomodulatory drugs for at least three months. In general, patients with neuroborreliosis are initially treated with ceftriaxone or penicillin G sodium, administered intravenously for 15–30 days, followed by an additional oral antibiotic schedule to complete three months of therapy.

BYS presents two relevant particularities that may be due to the particular features of *Borrelia burgdorferi* found in Brazil. First, it is common to find symptoms compatible with CFS, which include significant physical and mental fatigue persisting longer than 6 months that does not improve with rest, potentially involving myalgia, arthralgia, lympho-adenomegaly, neuro-cognitive symptoms, headache, sore throat, and sleep disturbance. Treatment of CFS is difficult, and CFS is not improved by antibiotic administration. In general, the use of analgesics and anti-depressive drugs, associated with psychological, nutritional and rehabilitation support, is recommended.

The second complication observed in patients with BYS is the presence of symptoms compatible with those found in autoimmune diseases. Following an acute infectious stage, patients sometimes develop arthritis; Raynauds phenomenon; cutaneous lesions similar to those observed in systemic lupus erythematosus; scleroderma; symptoms of sicca syndrome; myositis; thrombosis; vasculitis or allergic manifestations related to food, drugs, or insect bites. BYS patients often show the presence of autoantibodies in their serum, such as antinuclear antibodies (ANA), anti-Ro, anticardiolipin, antithyroglobulin, or hypergammaglobulinemia. Clinical symptoms of autoimmune disease do not respond to antibiotics. In some instances, it is necessary to employ corticosteroids, immunosuppressive agents and anti-allergic drugs. The pathogenesis of CFS and presentation of autoimmune symptoms are unknown. There is evidence of neuro-immune-endocrine axis activation, possibly triggered by “hidden” *Borrelia* or the presence of spirochete outer membrane surface proteins. In this respect, we demonstrated by PCR assays that Brazilian *Borrelia burgdorferi*, can be identified in the skin or blood of BYS patients for months or years after disease onset, even after antibiotic treatment of the patient, possibly due to the particular genetic and morphological adaptations of this bacterium to survive in mammalian hosts 4,7.

Unfortunately, despite many previous reports on BYS, the emerging zoonosis is still poorly understood and is confused with LD by most Brazilian physicians. Additionally, it is not accepted as a disease requiring compulsory notification by the Brazilian Health Surveillance System. The delay in the diagnosis BYS in its early infectious stage, when antibiotic treatment is effective, can give rise to serious, sometimes irreversible, clinical manifestations, which include chronic untreatable neuroborreliosis (encephalomyelitis, ataxia) and erosive arthritis. Furthermore, we question the frequency of patients treated as presenting idiopathic CFS or autoimmune diseases, when in fact they represent undiagnosed cases of *Borrelia burgdorferi* infection.

## AUTHOR CONTRIBUTIONS

Miziara, Serrano VA and Yoshinari N were responsible for the project planning and development, literature review, manuscript writing, grammar correction and English translation, and final project review.
